# Structural characterization and catalytic sterilization performance of a TiO_2_ nano‐photocatalyst

**DOI:** 10.1002/fsn3.1646

**Published:** 2020-06-01

**Authors:** Yue Yang, Chengli Guan, Shanyuan Chen

**Affiliations:** ^1^ Yangjiang Polytechnic Yangjiang China; ^2^ Guangdong Provincial Key Laboratory of Atmospheric environment and Pollution Control South China University of Technology Guangzhou China

**Keywords:** backpropagation neural network, *Candida albicans*, *Escherichia coli*, *Staphylococcus aureus*, sterilization performance, TiO_2_ nano‐photocatalyst

## Abstract

In view of the food safety and hygiene issues caused by pathogenic microorganisms, tetrabutyl titanate was used as a precursor for the preparation of a TiO_2_ nano‐semiconductor photocatalyst via the sol–gel process. The plate count method was then adopted to investigate the photocatalytic sterilization performance of the synthesized TiO_2_ nanoparticles toward *Escherichia coli*, *Staphylococcus aureus*, and *Candida albicans*. Subsequently, a backpropagation (BP) neural network model was developed to predict the photocatalytic sterilization performance. The photocatalyst was structurally characterized by the Brunauer–Emmett–Teller method for specific surface area determination, transmission electron microscopy, X‐ray diffraction, and X‐ray photoelectron spectroscopy. The results indicated that the prepared TiO_2_ nano‐photocatalyst was of high purity with a specific surface area of 76.5 m^2^/g and the particle size range 15–18 nm. The nanoparticles exhibited characteristic peaks corresponding to the oxide component Ti–O, hydroxyl group ˙OH and oxygen chemisorbed and presented an anatase‐dominated multiphase structure that enhanced the photocatalytic performance. UV irradiation at 254 nm produced better sterilization effects on *E. coli*, *S. aureus*, and *C. albicans*, with elimination rates after 30 min of reaction of 97.8%, 99.4%, and 93.6%, respectively. These results indicated that the TiO_2_ nano‐photocatalyst is a promising environmentally friendly catalyst with good sterilization performance. The constructed BP neural network also exhibited high training accuracy and good generalization ability, with correlation coefficients between the network‐predicted and experimental target values of 0.9789. These results support research on the intelligent processing of photocatalytic sterilization with TiO_2_ nanoparticles.

## Graphical abstract

The TiO_2_ nano‐photocatalyst was prepared and proved to be a promising environmentally friendly catalyst with good sterilization performance. The constructed BP neural network exhibited high training accuracy and good generalization ability and supported research on the intelligent processing of photocatalytic sterilization with TiO_2_ nanoparticles.

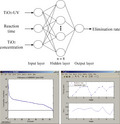

## 1 INTRODUCTION

Foodborne pathogenic diseases have become a major food safety problem and the most widespread hygiene issue globally (Dupont, 2007). Such diseases are mainly caused by bacteria Gabriel, Som, Madkour, Eren, and Tew (2007), the most common being salmonellae, pathogenic *Escherichia coli*, staphylococci, pathogenic streptococci, *Clostridium botulinum*, *Vibrio parahaemolyticus*, *Campylobacter jejuni*, and shigellae (Teunis, Takumi, & Shinagawa, 2004). In addition, pathogenic bacteria present in air‐conditioning systems and other confined spaces (various bacilli, molds, legionellae, pathogenic yeast *E. coli*, *Staphylococcus aureus*, and influenza viruses) also pose a direct threat to human health (Xu, She, & Xu 2003; Yan & Ding, 2011). Therefore, much emphasis has been placed on the research of antibacterial technologies and materials with broad‐spectrum sterilization effects and good sterilization performances.

Currently, common antibacterial agents can be classified into inorganic and organic agents (Zhang et al., 2016). Organic antibacterial agents, which mainly comprise aldehydes, alcohols, phenols, biguanides, iodophors, and surfactants, are the earliest known antibacterial materials. These agents come in wide variety and have low production costs; however, they possess several disadvantages including poor thermal resistance, high specificity, and a narrow antibacterial activity spectrum (Denis‐Rohr, Bastarrachea, & Goddard, 2015; Farah et al., 2015; Kenawy, Al‐Deyab, Shaker, El‐Sadek, & Khattab, 2009). In addition, their toxicity limits the food safety of organic antibacterial agents and promotes the development of antimicrobial resistance. On the other hand, inorganic antibacterial agents provide a higher level of safety and exhibit better thermal resistance, durability, and sustainability as well as a broad antibacterial activity spectrum. Thus, with the increasing demand for environmental quality, the market prospects of inorganic antibacterial materials are extremely promising (Lemire, Harrison, & Turner, 2013; Qin, Li, & Wang, 2017; Kalyani, Venkatraju, Kollu, Rao, & Pammi, 2015). However, inorganic antibacterial agents mainly comprise silver‐based formulations, which release endotoxins during the bacteria elimination process. Hence, they are currently unable to achieve the controllable sustained release of silver ions (Charles and Heinig, 1993; Cai, Hashimoto, Itoh, Kubota, & Fujishima, 1991).

In view of these issues, current research has been devoted toward creating novel antibacterial technologies (e.g., plasma sterilization, high voltage electrostatic water treatment, and photocatalysis) and materials (Xu et al., 2003). Following the report by Fujishima and Honda (1972) that TiO_2_ enables sustained redox reactions under photoirradiation in solar photovoltaic cells, research on the photocatalytic activity of TiO_2_ has been attracting increasing attention. Particularly, Carey, Lawrence and Tosine (1976) successfully applied TiO_2_ photocatalytic oxidation to the dechlorination and detoxification of polychlorinated biphenyls (PCBs) in water. Moreover, since the first discovery of the sterilization effects of UV‐activated TiO_2_ photocatalysts by Saito et al. (Saito, Iwase, Horie, & Morioka, 1992), the photocatalytic sterilization performance of TiO_2_ has led to significant research in many countries including China (Amezaga‐Madrid, Nevarez‐Mooriuon, Orrantia‐Borunda, & Miki‐Yoshida, 2002; Jing, Feng, Li, & Yu, 2013; Saito et al., 1992; Zhang, Su, Zhao, & Tan, 2008). This is because of the advantages of TiO_2_ nano‐photocatalytic antibacterial agents including a broad antibacterial activity spectrum, non‐toxicity, and nonproduction of secondary pollution and antimicrobial resistance. The combination of TiO_2_ with UV irradiation reported by Huang et al. (2000), and the combination of TiO_2_ with ultrasonic reported by Dadjour, Ogino, Matsumura, & Shimizu (2005) both deactivated the cells of *E. coli* by altering their cell wall, plasma membrane, and intracellular component structures. In separate studies, Sunada, Watanabe, and Hashimoto (2003) successfully applied TiO_2_ photocatalytic oxidation to eliminate *E. coli* in drinking water. Tian et al. (2009) reported that TiO_2_ displayed a good sterilization performance toward *S. aureus* and saccharomyces, while Li & Li (2015) adopted a TiO_2_‐supported catalyst in a sterilization experiment with *Bacillus subtilis* and *E. coli* and achieved an antibacterial circle diameter ≤25.8 mm. Huang, Xu, & Guo (2010) utilized TiO_2_ nanoparticles in the treatment of *Aeromonas hydrophila* and *Vibrio anguillarum* and achieved an elimination rate ≥98%. Other studies have also indicated that TiO_2_ photocatalysis can eliminate other microorganisms including various bacteriophages, yeasts and algae (Chamorn & Yasuyoshi, 2006; Zhao & Chen, 2008).

Although the photocatalytic performance of TiO_2_ nanoparticles is closely related to factors such as the crystal structure and specific surface area, reports on the relationship between the structure and catalytic sterilization performance of TiO_2_ nanoparticles are relatively scarce. In addition, as the photocatalytic sterilization effects of TiO_2_ are influenced by many factors, substantial debugging is required for the determination of the appropriate sterilization conditions. Thus, to conserve research resources, we have harnessed the intelligent nonlinear simulation advantages of neural networks and adopted them in the sol–gel process (Guan, Yang, & Chen, 2015; Yang, Yu, & Guan, 2018). This simple proposed method provided good uniformity for the synthesis of the TiO_2_ nano‐photocatalyst. Subsequently, the Gram‐negative (G^−^) bacterium *E. coli*, Gram‐positive (G^+^) bacterium *S. aureus*, and pathogenic yeast *Candida albicans* were selected as representative contaminant targets to investigate the crystal structure and photocatalytic sterilization performance of TiO_2_. In addition, a neural network was applied, for the first time, to predict the photocatalytic sterilization performance of TiO_2_ and thus, facilitate the rapid determination of optimum sterilization conditions.

## 2 MATERIALS AND METHODS

### 2.1 Chemicals

Brilliant green lactose bile broth, lactose bile fermentation broth, Sabouraud liquid medium, and plate count agar were purchased from Guangdong Huankai Microbial Sci. and Tech. Co., Ltd.; *E. coli* (GIM 1.355), *S. aureus* (GIM 1.221), and *C. albicans* (GIM 2.130) were purchased from the Guangdong Microbial Culture Collection Centre; low‐sodium salt was purchased from the Guangdong Salt Industry Group Co. Ltd. Anhydrous ethanol, nitric acid, and acetic acid were of AR grade, while tetrabutyl titanate was of CP grade. All the water used in the experiments was fresh ultrapure water.

### 2.2 Preparation of the TiO_2_ Nano‐photocatalyst

The TiO_2_ nano‐photocatalyst was prepared via the sol–gel process (Yang, Huang, Ye, & Chen, 2010) with tetrabutyl titanate (TBT; C_16_H_36_O_4_Ti) as the precursor. The detailed preparation procedure is as follows: 10 ml TBT was mixed with 30 ml anhydrous ethanol. Next, a solution mixture comprising 50 ml ethanol, 3 ml water, and 1.5 ml nitric acid was added dropwise into the TBT‐ethanol solution to obtain a light‐yellow gel. The gel was then mixed thoroughly for 12 hr and left to stand at room temperature for 24 hr. Subsequently, the gel sample was heated in a water bath at 80°C to evaporate the solvent. After drying, the gel was ball‐milled for 2 hr to obtain a fine powder, which was calcined in a temperature‐programmed muffle furnace, under inert N_2_ atmosphere, at 450°C for 2 hr. Finally, the calcined powder was milled again to produce the TiO_2_ nano‐photocatalyst, which was used in the subsequent experiments.

### 2.3 Characterization of the TiO_2_ nano‐photocatalyst

#### 2.3.1 Brunauer–Emmett–Teller (BET) analysis

BET analysis of the specific surface area (TriStar II 3020, Micromeritics Instrument Co.) was performed at the constant temperature 77 K using liquid nitrogen. Prior to testing, the samples were degassed at 573 K for 3 hr. The adsorbed volumes under different adsorption pressures were then measured, and the specific surface area of each sample was calculated using the BET equation.

#### 2.3.2 Transmission electron microscopy (TEM) Analysis

The microscopic structure and surface morphology of the photocatalyst were elucidated by TEM (JEM‐100CX II, JEOL Ltd.) using the following parameters: maximum accelerating voltage, 100 kV; magnification range, 100,000–200,000; maximum resolution, 3 Å; and maximum lattice resolution, 1.4 Å.

#### 2.3.3 X‐ray diffraction (XRD) analysis

XRD (D/MAX IIIA, Rigaku Co.) was performed under the following conditions: room temperature; Cu *K*_α_ X‐ray source, *K*_α_ radiation generated with a Cu target; tube voltage, 30 kV; tube current, 30 mA; scan range, 10–60°(2θ); and scan speed, 4°/min. The average particle size of the crystallites in the powdered sample was calculated using the Scherrer equation (Equation 1):

(1) d=0.89λ/βcosθ

where *β* is the full width at half maximum (FWHM) of the strongest diffraction peak of the sample, *λ* is the X‐ray wavelength, and *θ* is the diffraction angle.

#### 2.3.4 X‐ray photoelectron spectroscopy (XPS) analysis

XPS (VG Multilab 2000, Thermo Fisher Scientific) was next performed to analyze the elemental components and valence states on the catalyst surface. The following parameters were employed: energy resolution, 0.48 eV; imaging spatial resolution, 3 μm; minimum analysis area, 15 μm; X‐ray source, Mg K_α,_ hv = 1,253.6 eV; full spectrum range, 0–1,000 eV; and C 1s calibrated binding energy, 284.6 eV.

### 2.4 Evaluation of the sterilization performance of the TiO_2_ nano‐photocatalyst

#### 2.4.1 Preparation of the microbial suspensions

Based on the requirements of aseptic operations, the starter cultures of *E. coli*, *S. aureus*, and *C. albicans* were separately transferred onto sterile growth media on a clean bench and subsequently cultured in a biochemical incubator at 37°C for 24 hr. The *C. albicans* culture was cultured at 25°C to enable activation. The activated culture was then inoculated into the nutrient broth and incubated at constant temperature, with shaking, to reach the exponential growth phase. Prior to further use, each of the initially formulated microbial suspensions was subjected to a series of saline dilutions to concentrations in the range 1.0 × 10^5^–1.0 × 10^6^ cfu/ml (expected colony count, 30–300 cfu/plate).

#### 2.4.2 Influence of UV irradiation combined with the TiO_2_ nano‐photocatalyst (TiO_2_‐UV) on the elimination rate

For each of the prepared microbial suspensions, 0.01 g TiO_2_ nanoparticles were added to 100 ml suspension. The nanoparticle‐containing suspensions were then added to a series of plate cultures using the coating method, and photocatalysis was carried out under UV irradiation at 254 nm using a 16 W UV lamp. After 30 min of reaction, the plates were incubated in a biochemical incubator at 37°C (the *C. albicans* cultures were incubated at 25°C). The colonies were then counted using the plate count method and the sterilization performances of the proposed TiO_2_‐UV method with the three pathogens after 30 min reaction were investigated. Blank (reaction performed in the absence of TiO_2_ nanoparticles and UV irradiation) and control (reaction performed in the presence of either TiO_2_ nanoparticles or UV irradiation) groups were established for the experiment. In addition, to ensure data reliability, two independent parallel experiments were performed each time and each experiment was performed in triplicate to obtain the average values. The elimination rate of each type of microbe by the TiO_2_ nano‐photocatalyst was calculated using Equation (2):

(2) $$\begin{array}{cc} \text{Elimination rate}\left(\%\right) & =\left(\text{colony count of the blank group}\;-\;\text{colony count of the experimental group}\right)\\ & /\text{colony count of the blank group}\;\times \;100 \end{array}$$

#### 2.4.3 Influence of the reaction time on the elimination rate

Under UV irradiation at 254 nm, the photocatalytic reaction described in Equation 2 was performed to investigate the sterilization performances of the TiO_2_ nano‐photocatalyst (concentration: 0.1 g/L) toward the G^−^ and G^+^ bacteria and *C. albicans* at the reaction times 10, 20, 30, 40, 50, and 60 min. A blank group (reaction performed in the absence of TiO_2_ nanoparticles and UV irradiation) was established for this experiment, and the elimination rates were calculated using Equation 2.

#### 2.4.4 Influence of the TiO_2_ concentration on the elimination rate

The sterilization performances of the TiO_2_ nano‐photocatalyst at different concentrations (0.01, 0.1, and 1.0 g/L) toward the G^−^ and G^+^ bacteria and *C. albicans* (UV irradiation, 254 nm; reaction time, 30 min) were next investigated. A blank group (reaction performed in the absence of TiO_2_ nanoparticles and UV irradiation) was established for this experiment, and the elimination rates were calculated using Equation 2.

### 2.5 Intelligent optimization of the neural network

Backpropagation (BP) neural network algorithms account for 80%–90% of the total neural network algorithms used by researchers (Guan et al., 2015; Yang et al., 2018). In this study, experimental analysis was combined with a three‐layer BP neural network model constructed using MATLAB 7.1. The input layer parameters used in the model were the influencing factors of the experiments (TiO_2_‐UV, reaction time, and TiO_2_ concentration), while the output layer parameter was the elimination rate; the number of hidden layer parameters, as determined by simulation, was eight.

## 3 RESULTS AND DISCUSSION

### 3.1 Analysis of TiO_2_ characterization

#### 3.1.1 BET analysis

The BET results revealed that the specific surface area of the prepared TiO_2_ nano‐photocatalyst (76.5 m^2^/g) was higher than that of commercial P25 TiO_2_ nanoparticles prepared via the gas‐phase method (50 ± 15 m^2^/g) (Wen, Yi, Pei, & Xu, 2017). This marked increase implied enhanced catalytic activity of the prepared catalyst over that exhibited by P25 (Mulakov et al., 2020; Yang et al., 2010).

#### 3.1.2 TEM analysis

**FIGURE 1:**
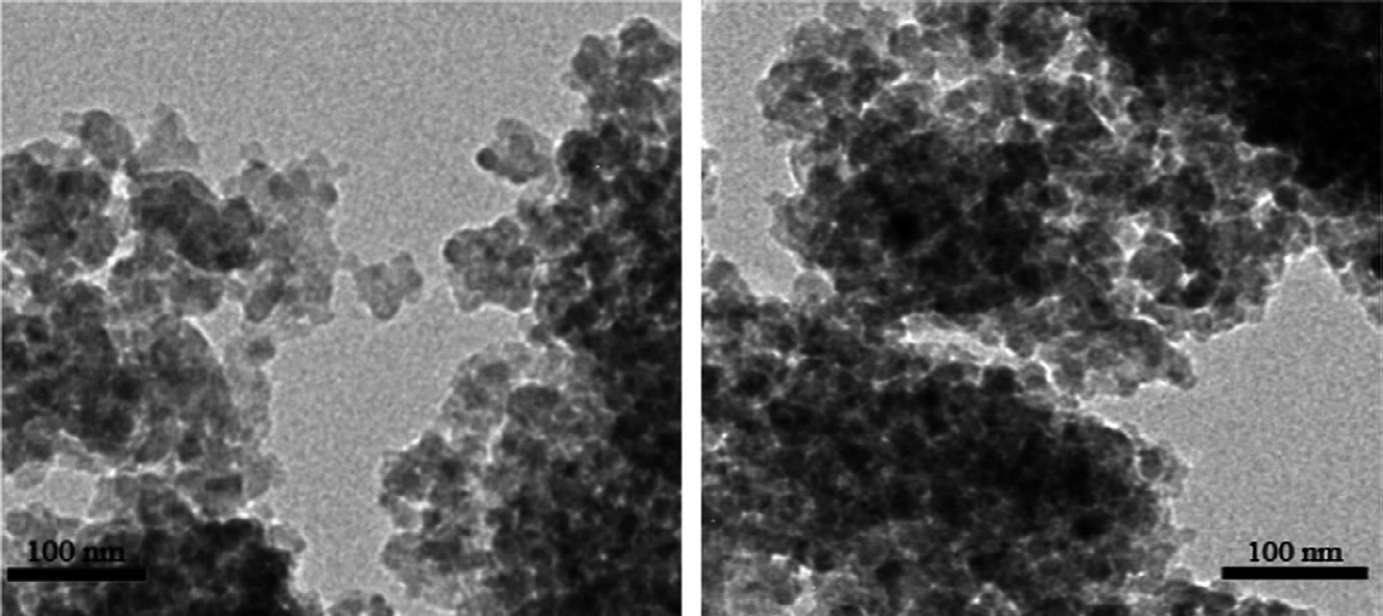
TEM image of TiO_2_

Figure 1 illustrates the transmission electron micrograph of the TiO_2_ nanoparticles prepared by calcination at 450°C. The TiO_2_ nanoparticles displayed a relatively uniform particle size in the range 15–18 nm, which was smaller than that of the P25 TiO_2_ nanoparticles. These results are consistent with the BET results and reveal that the TiO_2_ nanoparticles formulated in this study had higher specific surface areas than those of the P25 TiO_2_ nanoparticles.

#### 3.1.3 XRD analysis

**FIGURE 2:**
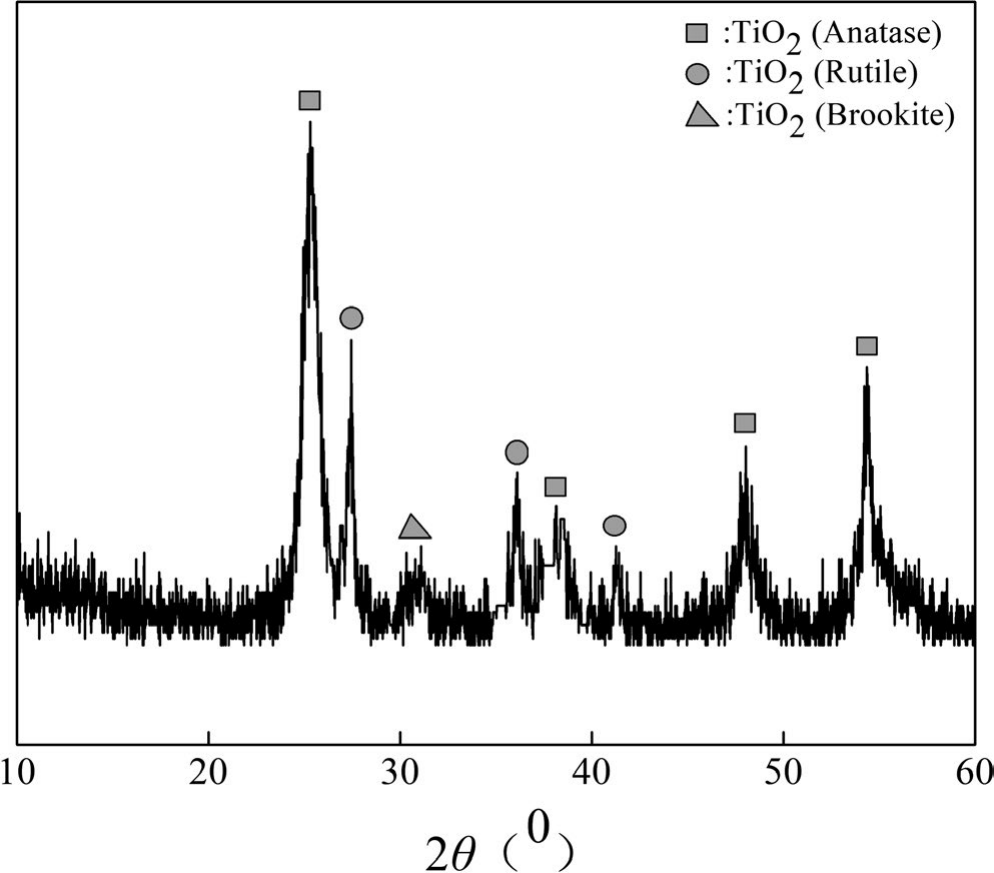
XRD pattern of TiO_2_

Figure 2 displays the XRD spectrum of TiO_2_ after calcination at 450°C for 2 hr. The peaks at 2θ = 25.32° (101), 38.12° (004), 48.06° (200), and 54.28° (105) corresponded to the characteristic peaks of the anatase crystal form, with the strongest diffraction peak at 2θ = 25.32° (Mulakov et al., 2020; Wu, Long, Cai, Chen, & Wu, 2010). Moreover, significant characteristic peaks of the rutile crystal form were observed at 2*θ* = 27.48° (110), 36.12° (101), and 41.26° (111), indicating the presence of a small amount of rutile crystals in the sample. And a weak characteristic peak of the brookite crystal appeared at 2θ = 30.81° (121) (Wu et al., 2010). These results revealed that the TiO_2_ nanoparticles possessed an anatase‐dominated mixed anatase–rutile–brookite crystal structure. Studies (Mulakov et al., 2020; Wang, Zhang, Zheng, & Shui‐Lin, 2014; Zhang, Zhou, & Lei, 2006) have revealed that anatase TiO_2_ has a higher catalytic activity than rutile or brookite TiO_2_, while TiO_2_ with a mixed crystal structure provides better catalytic effects than TiO_2_ with a single crystal structure. In addition, the average TiO_2_ particle size in the sample, preliminary calculated using Equation 1, was 16.9 nm, which is consistent with the TEM results.

#### 3.1.4 XPS analysis

**FIGURE 3:**
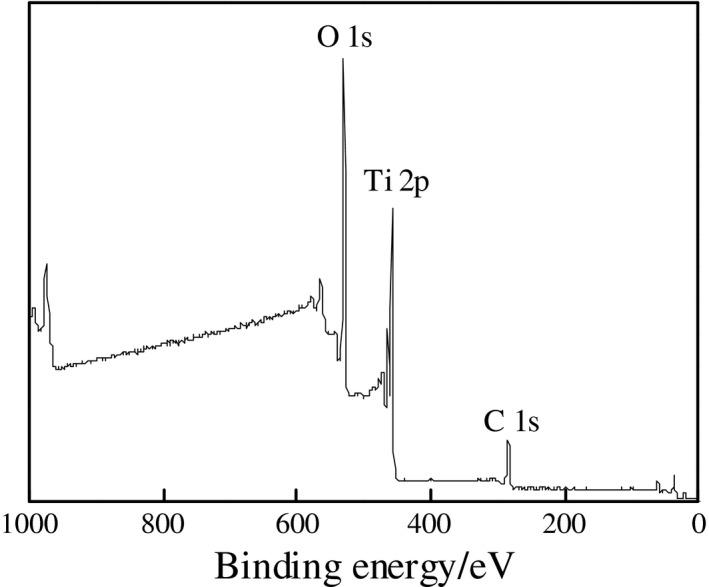
XPS spectrum of TiO_2_

Figure 3 displays the surface elemental compositions of the TiO_2_ nanoparticles determined by XPS analysis. The XPS full spectrum reveals that the main components of the TiO_2_ nanoparticles were Ti and O, with the atomic concentrations 18.75% and 48.56%, respectively (Ti:O ratio, 1:2.6). The difference between the experimental and theoretical (1:2) Ti:O ratios was attributed to the presence of other oxygen‐containing compounds, such as O_2_ gas and water, adsorbed onto the surfaces of the TiO_2_ nanoparticles, contributing to additional oxygen content besides the oxygen atoms within the TiO_2_ crystals. The results also revealed the presence of C, with C contents as high as 32.69%. This may have been caused by organic contamination either during the testing process or from the testing equipment. Using the high‐resolution O 1s XPS data of pure TiO_2_ for comparison, characteristic peaks of the oxide component Ti–O, hydroxyl group ˙OH, and oxygen chemisorbed were identified at the respective binding energies 530.6, 532.2, and 534.2 eV (Sanjines et al., 1994).

**FIGURE 4:**
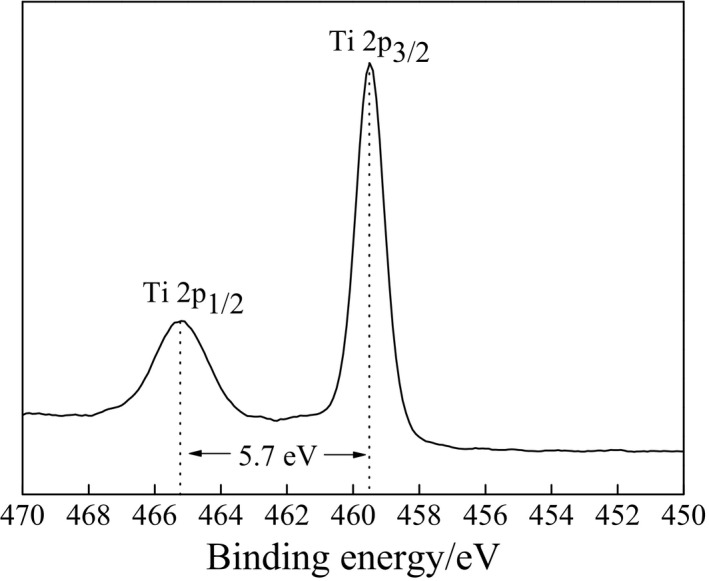
Ti 2p high‐resolution XPS spectrum of TiO_2_

Figure 4 presents the high‐resolution XPS spectrum of Ti 2p in pure TiO_2_ nanoparticles. It reveals that spin‐orbit coupling led to energy splitting of Ti 2p to Ti 2p_1/2_ and Ti 2p_3/2_, with the spectrum line exhibiting a symmetric Gaussian distribution. The electron binding energies of Ti 2p_3/2_ and Ti 2p_1/2_ were 459.5 and 465.2 eV, respectively, while the band gap was 5.7 eV, values that are consistent with results reported in the literature (Cao, Li, Zhang, & Wang, 2010). This suggests that Ti existed in the Ti^4+^ bound state, indicating a relatively high purity of the prepared TiO_2_ nanoparticles. The Ti 2p_3/2_ and Ti 2p_1/2_ electron binding energies in elementary Ti are 453.8 and 459.9 eV, respectively, with a band gap of 6.1 eV. Compared with these values, the Ti 2p_3/2_ energy of the TiO_2_ nanoparticles exhibited a chemical shift of 5.7 eV, attributed to the different chemical environment surrounding Ti.

### 3.2 Evaluation of the sterilization performance of the TiO_2_ nano‐photocatalyst

#### 3.2.1 Influence of UV‐TiO_2_ on the elimination rate

**FIGURE 5:**
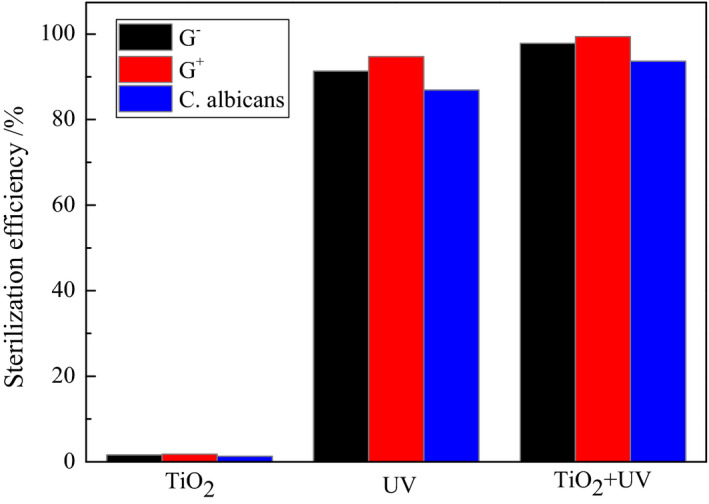
Effects of irradiation UV light

Figure 5 illustrates the sterilization performances of UV irradiation and the TiO_2_ photocatalyst when separately reacted with G^−^ and G^+^ bacteria and *C. albicans* for 30 min. The sterilization effect of the TiO_2_ nano‐photocatalyst was negligible in the absence of UV irradiation, while the sole use of UV irradiation on the G^−^ and G^+^ bacteria and *C. albicans* resulted in elimination rates of 91.3%, 94.7%, and 86.9%, respectively. Notably, when the TiO_2_ nano‐photocatalyst was combined with UV irradiation, the elimination rates of these three pathogens increased to 97.8%, 99.4%, and 93.6%, respectively. These results suggested a relationship between the UV irradiation and TiO_2_. Specifically, UV irradiation induced the generation of the electron–hole (e^−^–h^+^) pairs, which in turn oxidized the cell walls, cell membranes, and other components of the bacteria and yeast cells, thereby leading to bacterial and fungal deactivation and even death (Tian et al., 2009). In addition, XPS analysis revealed that the TiO_2_ samples prepared in this study contained ˙OH radicals with activation energies ≤402.8 MJ/mol. This enabled the rapid and effective decomposition of the organic cellular components and organic nutrients (necessary for growth and reproduction), thereby causing protein denaturation and ultimately death.

#### 3.2.2 Influence of the reaction time on the elimination rate

**FIGURE 6:**
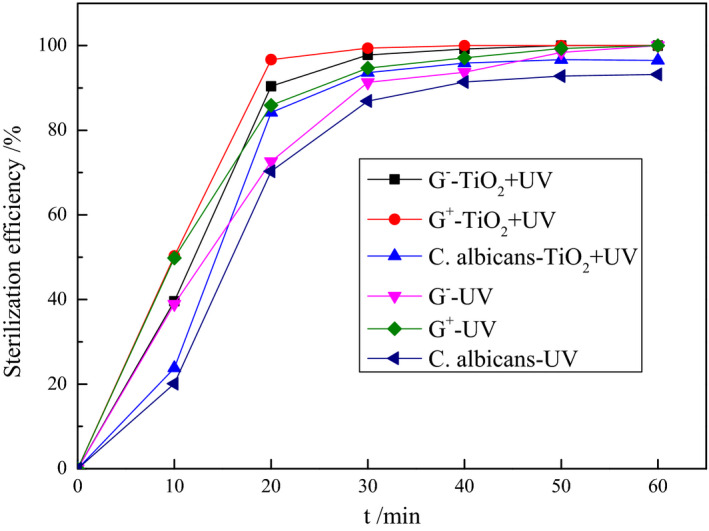
Effects of photocatalytic time

Figure 6 displays the sterilization performances of 0.1 g/L TiO_2_ nano‐photocatalyst when separately reacted with G^−^ and G^+^ bacteria and *C. albicans* at different reaction times under UV irradiation. The TiO_2_ elimination effects on the two types of bacteria and *C. albicans* can be divided into three phases: In the first phase, the first 20 min of the reaction, the photocatalytic elimination rates of the G^−^ and G^+^ bacteria by TiO_2_ rapidly increased to ≥90%, while the elimination rate of *C. albicans* increased to 84.2%. In the second phase, between 20 and 40 min reaction, the photocatalytic elimination rates of the G^−^ and G^+^ bacteria and *C. albicans* increased slowly until they reached 99.2%, 100%, and 95.9%, respectively, after 40 min. In the third phase, when the reaction time exceeded 40 min, the photocatalytic elimination rates of the bacteria and *C. albicans* leveled off at 100% and 97%, respectively. Under sole UV irradiation, without the presence of the photocatalyst, the elimination rates of both bacteria only exceeded 90% after 30 min and gradually leveled off at 100% after 50 min reaction. For *C. albicans*, the elimination rate only exceeded 90% after 40 min and eventually leveled off at ~93%. Based on the XRD data, the particle size of the prepared TiO_2_ nano‐photocatalyst was 16.9 nm. These ultrafine TiO_2_ nanoparticles could be rapidly and completely adsorbed onto the pathogen surfaces, thereby enabling rapid photocatalytic activity upon UV irradiation. In this case, the generated e^−^–h^+^ pairs and ˙OH radicals could penetrate and damage structures such as the cell walls within the pathogen through oxidation, thereby achieving good sterilization effects in a relatively short time.

#### 3.2.3 Influence of the TiO_2_ concentration on the elimination rate

**FIGURE 7:**
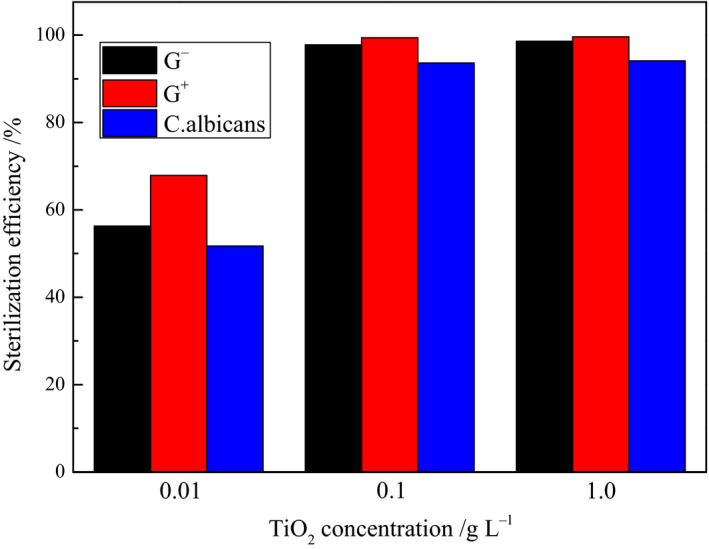
Effects of TiO_2_ concentration

Figure 7 illustrates the influence of different TiO_2_ nano‐photocatalyst concentrations on the sterilization performances toward the G^−^ and G^+^ bacteria and *C. albicans*. The elimination rates of the three pathogens increased with increasing TiO_2_ concentration. When the TiO_2_ concentration was 0.01 g/L, the elimination rates of the G^−^ and G^+^ bacteria and *C. albicans* were 56.3%, 67.9%, and 51.7%, respectively. This indicated that an excessively low concentration of TiO_2_ led to the generation of fewer active sites (e^−^–h^+^ pairs and ˙OH radicals) upon UV irradiation. In addition, these sites were not effectively utilized, thereby leading to lower elimination rates. These results suggested that a higher TiO_2_ concentration would produce more active oxidants and thus, enhanced elimination rates. In fact, the increase in TiO_2_ concentration, from 0.01 to 0.1 g/L, led to a significantly positive correlation between the elimination rate and TiO_2_ concentration, and the elimination rates of the G^−^ and G^+^ bacteria and *C. albicans* increased to 97.8%, 99.4%, and 93.6%, respectively. However, when the TiO_2_ concentration was further increased to 1.0 g/L, the elimination rates of the three types of representative microbes merely increased by 0.8%, 0.2%, and 0.5%, respectively, when compared to the elimination rates achieved with a TiO_2_ concentration of 0.1 g/L. An excessively high TiO_2_ concentration increases the solution turbidity and causes the light scattering to gradually exert a dominant effect on the light transmittance of the solution. This weakening in the light intensity in the solution in turn decreases the quantum efficiency, thereby leading to the absence of a significant correlation between the elimination rate and TiO_2_ concentration. This was reflected in the poor increase in the microbe elimination rates at the highest TiO_2_ concentration.

### 3.3 Intelligent optimization of the neural network

**FIGURE 8:**
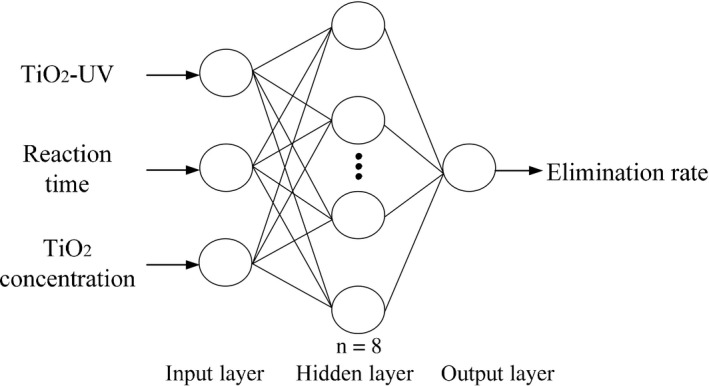
BP neural network structure

Using *E. coli* as the microbial species, a BP neural network for photocatalytic sterilization by TiO_2_ nanoparticles was next constructed (Figure 8). The training accuracy of the model was set at 0.001 and the functions used in the model were as follows: transfer function of the output layer: pureline; transfer function of the hidden layer: tansig; training function: trainbfg; learning function: learngd; performance function: mse. In addition, to reduce the differences between the different parameters and enhance the operation accuracy of the network, the mapminmax function in the Neural Network Toolbox in MATLAB was used for the standardization of the experimental data.

**FIGURE 9:**
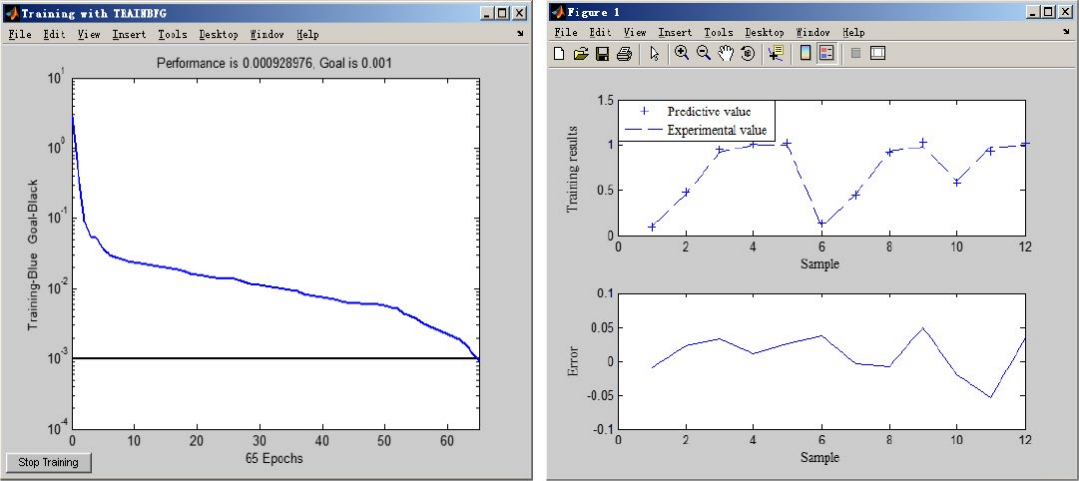
Training process and results of BP neural network

In this study, 17 sets of experimental data were randomly allocated to the training (Kalyani et al., 2015) and prediction (five sets of data) datasets. Figure 9 illustrates the training processes and results when the BP neural network was run. It reveals that the constructed BP neural network converged rapidly with the specified training accuracy limit and the system output values were close to the experimental values, with all errors being within ±5%. This revealed that the constructed neural network provided a good approximation of the variation patterns of the training dataset and could reflect the sterilization performances of the TiO_2_ nano‐photocatalyst toward *E. coli* under different parameter conditions.

**FIGURE 10:**
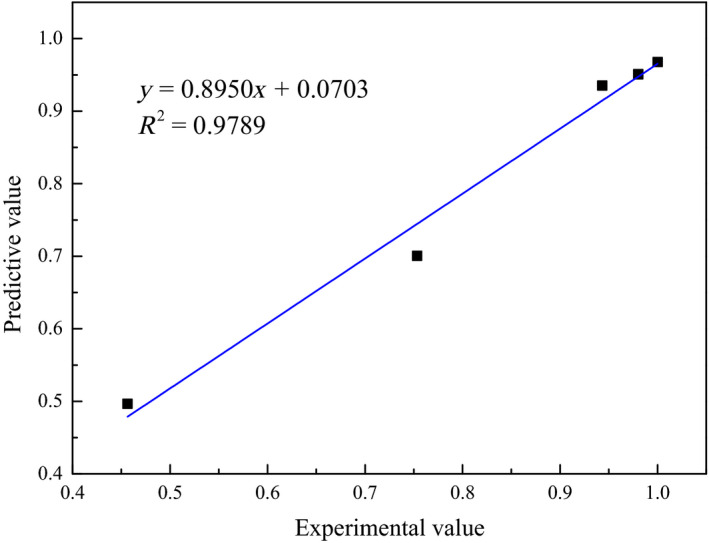
Comparison of predictive values and experimental values

To evaluate the generalization ability of the network, five sets of prediction data were used as the test data (Figure 10). The results indicated a close match between the network‐predicted and actual values with relatively small errors, demonstrating that the network had a good generalization ability and could provide good estimates from untrained data. Therefore, we concluded that the BP neural network constructed in this study can be used to predict the oxidative sterilization effects of the TiO_2_ nano‐photocatalyst on *E. coli* under different parameters. This contributes to resource and cost savings and thus increases the potential of this method for real application.

## 4 CONCLUSIONS

In this study, the sol–gel method was adopted to prepare a TiO_2_ semiconductor photocatalyst. When the prepared catalyst was calcined at 450°C, nano‐sized particles with an anatase‐dominated mixed crystal structure and good photocatalytic effects under UV irradiation were obtained. Subsequently, the sterilization performances toward the pathogenic bacteria *E. coli* and *S. aureus* and the pathogenic yeast *C. albicans* under the influence of different factors (TiO_2_‐UV, reaction time, and TiO_2_ concentration) were investigated. Under UV irradiation combined with 0.1 g/L TiO_2_, the elimination rates of *E. coli*, *S. aureus*, and *C. albicans* after 30 min reaction reached 97.8%, 99.4%, and 93.6%, respectively. However, after 40 min reaction, the elimination rates of the two bacterial species stabilized at 100%, while the elimination rate of *C. albicans* stabilized at ~97%. The prepared nano‐photocatalyst is non‐toxic, does not generate secondary pollution, and produces good sterilization effects toward the representative G^−^ and G^+^ bacteria and pathogenic yeasts. Thus, this proposed technology shows great potential for application and popularization in the field of food hygiene. A BP neural network was employed to achieve both energy and resource savings. The sample data training and prediction results indicated that the network presented a good generalization ability, with a correlation coefficient of 0.9789 between the network‐predicted and experimental values. This demonstrates the ability of the network to well predict the photocatalytic sterilization effects of the TiO_2_ nanoparticles under different parameter conditions. Therefore, this model is of great engineering significance and shows great potential for further application and popularization in the relevant fields.

## CONFLICT OF INTEREST

The authors declare that they do not have any conflict of interest.

## ETHICAL APPROVAL

This study does not involve any human or animal testing.
